# Oligomerization of Hsp70: Current Perspectives on Regulation and Function

**DOI:** 10.3389/fmolb.2019.00081

**Published:** 2019-09-04

**Authors:** Jade E. Takakuwa, Nitika, Laura E. Knighton, Andrew W. Truman

**Affiliations:** Department of Biological Sciences, The University of North Carolina at Charlotte, Charlotte, NC, United States

**Keywords:** Hsp70, molecular chaperones, dimerization, oligomerization, proteostasis

## Abstract

The Hsp70 molecular chaperone in conjunction with Hsp90 and a suite of helper co-chaperones are required for the folding and subsequent refolding of a large proportion of the proteome. These proteins are critical for cell viability and play major roles in diseases of proteostasis which include neurodegenerative diseases and cancer. As a consequence, a large scientific effort has gone into understanding how chaperones such as Hsp70 function at the *in vitro* and *in vivo* level. Although many chaperones require constitutive self-interaction (dimerization and oligomerization) to function, Hsp70 has been thought to exist as a monomer, especially in eukaryotic cells. Recent studies have demonstrated that both bacterial and mammalian Hsp70 can exist as a dynamic pool of monomers, dimer, and oligomers. In this mini-review, we discuss the mechanisms and roles of Hsp70 oligomerization in Hsp70 function, as well as thoughts on how this integrates into well-established ideas of Hsp70 regulation.

## Introduction

In situations where proteins may lose structural integrity in response to stress, Hsp70 can rebind and refold “client” proteins restoring their cellular function (Rosenzweig et al., 2019). If refolding of a client protein cannot be achieved, Hsp70 assists in the transport of proteins to the proteasomal degradation machinery. The essential nature of Hsp70 has meant that minimal amino acid sequence variation has accumulated throughout evolutionary history and Hsp70 can be found in various forms from prokaryotes to eukaryotes. Although there is only a single Hsp70 expressed in bacteria (DnaK), other organisms express several isoforms. The budding yeast *Saccharomyces cerevisiae* possess a total of 11 Hsp70 paralogs; 4 semi-redundant cytosolic forms (Ssa1-4), 3 located in the mitochondria and 1 ER-specific form (Kar2) (Kominek et al., 2013). In contrast, Humans express 13 Hsp70 gene products that vary in activity, expression, and cellular localization (Kabani and Martineau, 2008).

Despite the large number of human isoforms, two forms predominate. Hsc70 is constitutively expressed and provides essential housekeeping function, while the inducible form Hsp70 is increased in response to a rise in misfolded proteins. The structure of Hsp70 is comprised of three major domains; an N–terminal ATPase domain (NBD), a 44-KDa structure that is involved in the binding of Hsp70 and hydrolysis of ATP, a 28 kDa Substrate binding domain (SBD) that binds unfolded and misfolded clients, and a C-terminal region containing leucine rich EEVD motif, essential for client refolding. The NBD and SBD are connected via a flexible linker domain that transmits structural re-arrangements brought about by the hydrolysis of ATP to the SBD, allowing client folding (Rosenzweig et al., 2019).

While much of the previous work on Hsp70 have focused on regulatory paradigms such as expression of Hsp70 isoforms in cells, co-chaperone function and ATP binding and hydrolysis, it is clear that Hsp70 can be regulated in other ways. Recently, several groups have identified regulatory post-translational modifications (PTMs) on Hsp70 that include acetylation, phosphorylation, ubiquitination, oxidation, and AMPylation (Truman et al., 2012; Wang et al., 2012; Truttmann et al., 2016; Nitika and Truman, 2017; Zemanovic et al., 2018). Although work is ongoing to match function to specific PTMs on Hsp70, it is clear that combination of PTMs present at any one time on Hsp70 (the “chaperone code”) do play an important role in the *in vivo* and *in vitro* function of Hsp70 (Cloutier and Coulombe, 2013; Nitika and Truman, 2017). Here we summarize the current knowledge on Hsp70 oligomerization, including potential mechanisms that regulate the formation of Hsp70 oligomers as well as their functional consequences. Finally, we discuss how these different aspects of Hsp70 regulation may be tied together.

## Regulatory Mechanisms that Control Formation of Hsp70 Oligomerization

### Nucleotide Bound Status of Hsp70

The bacterial Hsp70 homolog DnaK exists primarily in monomeric form, although mixtures of monomers, dimers, trimers and oligomers have been observed *in vitro* and *in vivo* (Schonfeld et al., 1995; Thompson et al., 2012; Sarbeng et al., 2015). As with all Hsp70s, the binding and hydrolysis of ATP to ADP promotes structural rearrangements. While DnaK can form dimers while bound to ATP, the majority of DnaK is monomeric in the presence of ATP (Thompson et al., 2012; Sarbeng et al., 2015; Trcka et al., 2019). In the presence of ADP, DnaK exhibits an array of dimers, trimmers, and higher weight oligomers. The T199A mutant of DnaK, which is able to bind but not hydrolyze ATP exists primarily as a monomer, further supporting the relationship nucleotide binding of Hsp70, and self-association (Sarbeng et al., 2015).

The role of nucleotide binding in formation of mammalian Hsp70 oligomers remains unclear, with conclusions differing based on whether the experiments were done on intact cells, cell lysates, or purified protein. Earlier studies using cell lysates clearly demonstrated that ATP promotes the dissociation of oligomers of the ER-localized Hsp70 BiP, bovine Hsp70, and its constitutive counterpart, Hsc70 (Palleros et al., 1991; Carlino et al., 1992; Kim et al., 1992; Benaroudj et al., 1996; Angelidis et al., 1999). A more recent study on purified chaperone protein suggests that ATP is required for at least the initial formation of Hsp70 dimers with the ATP-bound conformation of Hsp70 necessary for interaction with another Hsp70 molecule (Trcka et al., 2019).

### Exposure to Cell Stress

Hsp70s exist as a mixture of both monomers and higher order states suggesting a regulated mechanism of Hsp70 self-association. Several cellular stressors, such as hydrogen peroxide-induced oxidative stress and stationary phase stress do not affect oligomerization of DnaK, however increases in temperature increased the levels of oligomerization (Thompson et al., 2012). Heat stress itself may be the trigger for oligomerization, since during heat stress the ADP/ATP ratio is altered to favor ADP, and the expression of DnaK is heightened, both factors that increase the proportion of oligomers in DnaK. In mammalian cells, heat stress encourages formation of the dimeric state of the constitutive Hsc70; in contrast, the heat-inducible Hsp70 which exists primarily as a dimer, remains unchanged by heat (Angelidis et al., 1999). Interestingly, heat stress induces tyrosine and serine phosphorylation on mammalian Hsp70 and may be connected to altered oligomerization (Maher and Pasquale, 1989; Kim et al., 2002).

### Post-translational Modification of Hsp70

There is growing evidence that a range of post-translational modifications (PTMs) such as phosphorylation, acetylation, methylation, ubiquitination, and SUMOylation all play a role in the activity, and specificity of chaperones and co-chaperones (Cloutier and Coulombe, 2013; Mollapour et al., 2014; Nitika and Truman, 2017; Sager et al., 2019). Tyrosine phosphorylation of Hsp70 regulates both its activity and translocation to the nucleus (Knowlton et al., 2000). Importantly, it is also associated with multimeric forms of Hsp70 in cancer cells, although the residues involved and regulatory mechanisms have not been fully studied (Dutta et al., 2013). Mammalian Hsp70 purified from two different sources (*E. coli* and Sf9 insect cells) varied in both PTM content and monomer-dimer population (Morgner et al., 2015). The additional modifications detected in Sf9-purified Hsp70 included seven lysine acetylation sites in addition to a phosphorylation hotspot/phosphosite T504, which stabilizes the dimeric form of Hsp70 (Morgner et al., 2015).

Post-translational modifications also play a role in the oligomerization of the ER-resident Hsp70, BiP. BiP undergoes both phosphorylation and ADP ribosylation, modifications which are only found on the dimeric and oligomeric form. In the immune system, transport and assembly of the heteromeric immunoglobulin molecule (H_2_L_2_) is controlled by BiP (Lee et al., 1999). BiP that is bound to non-transported immunoglobulin heavy chains exists in post-translationally modified oligomeric form or unmodified monomeric form, and can interconvert between the two forms (Freiden et al., 1992; Gaut, 1997). Glucose starvation or the increase of non-transported heavy chains increases the synthesis of BiP and decreases its post-translational modification, leading to a greater concentration of monomers and the disappearance of the oligomeric form (Freiden et al., 1992). As with Hsp70, purifying BiP from a host that does not allow phosphorylation (e.g., *E.coli*) produces BiP that is primarily monomeric with no evidence of oligomers, while BiP purified from bovine extracts exists as an array of monomers, dimers, trimers, and oligomers (Carlino et al., 1992; Blond-Elguindi et al., 1993).

## Structural Requirements for Hsp70 Oligomerization

Early studies pinpointed the SBD as essential for oligomeric and dimeric formation (Benaroudj et al., 1997; Angelidis et al., 1999; Fouchaq et al., 1999). The C-terminal domain was determined to be the only determinant of the self-association of Hsp70, although a head-to-tail interaction of the C-terminal domain and the N-terminal domain could not be ruled out (Benaroudj et al., 1997). Using a 60 kDa fragment of Hsc70 and a C-terminal mutant, the interface of oligomerization was narrowed down to the 17-kDa domain from residues 385 to 540 on Hsc70 (Fouchaq et al., 1999). More recent higher resolution studies have employed substrate binding domain mutants and truncations to pinpoint the role of the C-terminal domain of Hsp70 in dimerization. Removal of the C-terminal section of Hsp70 results in a purely monomeric state; in contrast Hsp70 fragments lacking part the NBD exist almost exclusively as a population of oligomers, forming dimers, trimers, and tetramers (Aprile et al., 2013; Marcion et al., 2015).

**Figure 1:**
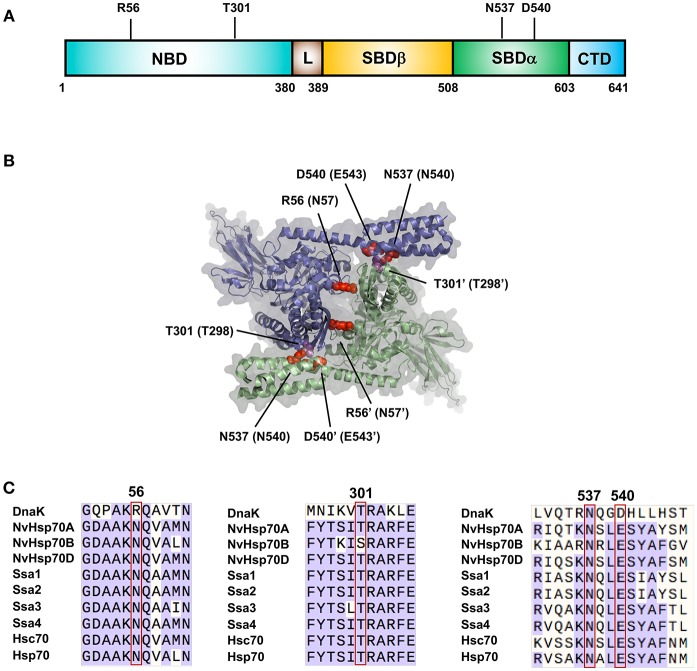
Important residues for Hsp70 dimerization. **(A)** Location of residues important for DnaK dimerization. **(B)** Structure of the DnaK dimer based on PDB entry 4JNE (Qi et al., 2013). The two DnaK protomers are colored purple and green. Amino acids important for dimerization of DnaK are labeled with equivalent Hsp70 residues in parentheses in red/magenta. **(C)** Sequence alignments of major Hsp70 isoforms (bacterial DnaK, Nematostella vectensis Hsp70 **(A–C)**, yeast Ssa1-4 and human isoforms Hsp70 and Hsc70) showing regions critical for dimerization.

The understanding of Hsp70 family oligomerization has been greatly aided by the generation of a high-resolution crystal structure of a dimer of DnaK [PDB: 4JNE (Qi et al., 2013)]. This structure contains two ATP bound protomers in anti-parallel arrangement (Figure 1A). Two dimer interfaces are apparent from the structure, NBD-NBD' and NBD-SBD'. On the NBD-NBD' interface, hydrogen bonding is observed between R56 and Q272', Q28 and R345'. On the NBD-SBD' interface, bonding between N537-Q277'/A303', D540-T301' and R536-A303/T301 (Qi et al., 2013). Mutation of any key residues at the dimerization sites to alanine (R56A, T301A, N537A, and N540A), produced DnaK significantly impaired for ability to dimerize. Two residues important for dimerization in DnaK (N537 and D540) are also conserved in human Hsp70 (Figure 1B). Mutations of these equivalent residues (N540A and E543A) individually reduced dimerization, while the double mutant N540A-E543A was completely unable to form a dimer (Trcka et al., 2019) suggesting biological conservation of dimerization throughout the Hsp70 family.

The propensity for Hsp70 to oligomerize may be evolutionarily well-conserved. Sequence analysis of Hsp70 family members identified a subset of co-evolving residues that was not compatible with a monomeric structure (Malinverni et al., 2015). This subset of residues could be divided into two sets, one set associated with the SBD alpha-lid to the NBD of another monomer, and the other involved in NBD-NBD interactions. The decreasing importance of dimer interactions in eukaryotes as well as the similarity between the arrangement of the Hsp70 homodimer and the Hsp70/Hsp110 heterodimer points to possible evolution of the Hsp70/Hsp110 heterodimer from a prokaryotic Hsp70 homodimer (Malinverni et al., 2015).

## Functional Consequences of Hsp70 Oligomerization

### Intrinsic Chaperone Function

Although ATP hydrolysis can impact oligomer formation, the reverse does not appear to be true. Mutation of key residues for dimerization in DnaK (N537 or D540) or in human Hsp70 (N540A, E543A) does not significantly impact the *intrinsic* ATPase activity of Hsp70 (Sarbeng et al., 2015; Trcka et al., 2019). Interestingly, these mutations do substantially decrease both peptide binding and refolding capabilities, key markers of Hsp70 chaperone function. In addition, the oligomeric form of DnaK bound to ADP exhibits hindered chaperone activity, showing an inability to refold denatured luciferase at the same rates as monomeric DnaK bound to ATP (Thompson et al., 2012). Oligomeric DnaK was still able to bind to denatured luciferase via its substrate binding domain, indicating that the dimeric interface does not block all substrate binding areas on the protein (Thompson et al., 2012). Both crosslinked ADP-bound DnaK and ATP-bound DnaK were able to protect luciferase from terminal heat denaturation, also known as holdase activity (Thompson et al., 2012). The impeded ability to perform protein refolding may lie in defective interactions with co-chaperones while in an oligomeric or dimeric state (see below).

### Interactions With Co-chaperones and Clients

Co-chaperone proteins are critical for regulating the activity and specificity of Hsp70 (Kampinga and Craig, 2010). Given that many co-chaperones bind to the N-terminus of Hsp70 near the proposed NBD-NBD dimerization interface, it is unsurprising that Hsp70 dimerization, and oligomerization has been found to alter interaction with co-chaperones. In bacteria, mutation of the DnaK dimerization interface decreased DnaK-DnaJ affinity resulting in a corresponding decrease in DnaJ-mediated ATPase stimulation (Sarbeng et al., 2015). This effect appears to be conserved in mammalian cells, where mutation on the same residues of Hsp70 triggered a corresponding loss of Hsp40, Bag1, and CHIP interaction (Trcka et al., 2019). While some co-chaperone interactions are enhanced post-Hsp70 dimerization, other co-chaperones such as Tomm34 can promote dimer dissociation (Trcka et al., 2019). Taken together these studies offer hints into how monomer-dimer-oligomer ratios may be controlled in the cell.

Few studies have analyzed the impact of Hsp70 dimer formation on client binding. Early work on Hsc70 demonstrated a capability to oligomerize during the removal of clathrin from coated vesicles during receptor-mediated endocytosis (Schmid and Rothman, 1985; Chappell et al., 1986). Hsc70 formed a complex with the partially bound triskelion and remained bound until all attachment to the clathrin cage was severed (Schmid and Rothman, 1985). Hsc70 purified from calf brains was found to consist of monomers and dimers and trimers with trimers localized to the vertices of clathrin triskelia (Schlossman et al., 1984; Heuser and Steer, 1989). In the presence of ATP, Hsc70 was monomeric and did not bind to clathrin triskelia, indicating that ATP modulates the removal of clathrin by stimulating Hsc70 monomerization. A proposed cycle for Hsc70 uncoating of clathrin involves three monomers of Hsc70, all bound to ATP, attaching to domains on the clathrin triskelia. Binding triggers hydrolysis of ATP which leads to a conformational change that favors polymerization, leading to a trimeric formation. Trimeric Hsc70 causes clathrin to separate from the greater clathrin cage surrounding the vesicle, and ATP rebinding promotes monomerization of Hsc70, which dissociates from the triskelia to re-enter the cycle (Schmid and Rothman, 1985). More recently, structural studies have characterized an Hsp90-Hsp70-HOP-GR complex that contains two Hsp70s in a dimer (Morgner et al., 2015; Blair et al., 2019). While it is hard to make broad inference from this single static structure, it does suggest that Hsp70 dimerization is a pre-requisite for binding and folding at least a selection of client proteins.

### Hsp70 Oligomerization and Cancer

Molecular chaperones play an important role in tumor initiation and progression (Goloudina et al., 2012; Sherman and Gabai, 2015; Calderwood and Gong, 2016; Joshi et al., 2018; Shevtsov et al., 2018; Wang et al., 2019). Hsp70 is upregulated in the plasma membrane of tumor cells and immobilizing Hsp70 decreases endocytic activity, indicating that such activity might depend on oligomerization or dimerization (Nimmervoll et al., 2015). Clusters of surface-bound Hsp70 were found to include fairly equal concentrations of monomers, dimers, and oligomers (Nimmervoll et al., 2015). Interfering with the possible sources of oligomer interaction, such as the inter-domain linker and the C-terminal of the helical lid subdomain, as well as omitting the NBD domain, decreased endocytic activity (Nimmervoll et al., 2015).

### Conclusion and Future Perspectives

Growing evidence from numerous studies on Hsp70 suggests that dimerization and higher-state oligomerization have important functional implications, affecting interactions with co-chaperones and client binding complexes. In contrast to other chaperone proteins such as Hsp90 and Hsp40 whose constitutive dimerization is essential for their cellular function, Hsp70 self-association is clearly required for only a subset specific set of chaperone functions and is dispensable for cell viability. If a subset of Hsp70 needs to exist in a self-associated state, what then is the initiating signal that regulates the monomer to oligomer transition? It is clear that PTMs on can alter propensity for dimerization (Morgner et al., 2015). Studies from our group and others on the chaperone code show that PTMs also alter Hsp90/Hsp70-co-chaperone binding *in vitro* and *in vivo* (Mollapour et al., 2011; Mollapour and Neckers, 2012; Truman et al., 2012; Dunn et al., 2015; Wolfgeher et al., 2015; Woodford et al., 2016; Dushukyan et al., 2017; Nitika and Truman, 2017; Weissman et al., in press). Our working model is that conditions that alter the chaperone code such as heat shock, DNA damage, nutrient availability, cell cycle stage and even age of a cell has the potential to alter both co-chaperone binding and oligomerization. This oligomerization may in turn may fine-tune the Hsp70 interactome, which is dynamic and has been shown to be rapidly altered in response to change in the chaperone code and exposure to cell stress (Mollapour et al., 2011; Truman et al., 2012, 2015a,b; Woodford et al., 2016).

**Figure 2:**
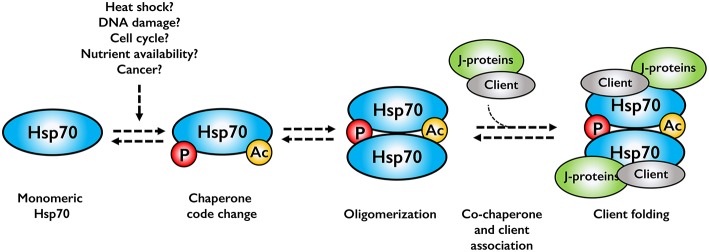
Assembly line model for Hsp70 oligomerization. Cell stresses alter the chaperone code, which promotes Hsp70 oligomerization. Co-chaperones and associated clients bind to the Hsp70 oligomer, forming an active chaperone complex.

Although the research on Hsp70 oligomerization is in its infancy, oligomerization may serve several purposes in protein quality control. Hsp70 oligomers may function as storage oligomers, dissociating, and becoming active upon cellular stress. Although feasible, this would imply that the dimer and oligomer forms are completely inactive, which is clearly not the case. A more reasonable model is a chaperone assembly line, where cell stress alters the chaperone code, leading to oligomerization. This in turn would promote association of co-chaperones and client to allow client folding (Figure 2). This model is indicated in the literature, but clearly cannot not hold true for all clients as prevention of dimerization would lead to cell death, a phenotype not experimentally observed, at least in bacteria.

The sequence similarity between Hsp70 isoforms raises an interesting question: can Hsp70 isoforms heterodimerize and if so, what does this mean for functionality? A precedent already exists with yeast Ssa1 forming a heterodimer with the co-chaperone Sse1 (Shaner et al., 2004; Andreasson et al., 2008). The yeast Ssa1-4 proteins display high conservation in the proposed regions for dimerization and theoretically could interact with one another. Although thought to be functionally redundant it is now clear that the Ssa1 isoforms have distinct cellular roles (Lotz et al., 2019). It would be interesting to analyze Ssa isoform interaction, especially under conditions where all four isoforms are expressed such as heat shock. Understanding the structural and cellular requirements for Hsp70 self-association may have broad-reaching implications in manipulating chaperone function, particularly in Hsp70-mediated pathologies such as cancer.

## Author Contributions

JT, and N wrote the initial drafts of the manuscript. LK created Figure 1 and AT contributed to figure design and edited the manuscript.

### Conflict of Interest Statement

The authors declare that the research was conducted in the absence of any commercial or financial relationships that could be construed as a potential conflict of interest.

## Abbreviations

HSP: Heat Shock Protein
NBD: Nucleotide-binding domain
SBD: Substrate-binding domain
HS: Heat Shock.
